# Supplementary figures and images for: Spatial transcriptomic profiling of decalcified murine musculoskeletal samples via Xenium Prime 5K
Source: JBMR Plus. 2026 Apr 14;10(6):ziag070. doi: 10.1093/jbmrpl/ziag070 (PMC13160414; doi:10.1093/jbmrpl/ziag070)

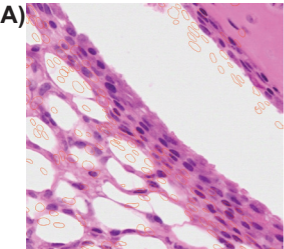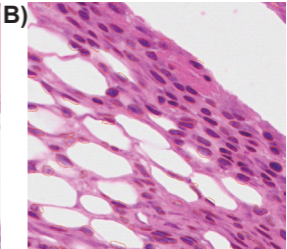

Supplement: ziag070_Supplemental_Files [file ziag070_supplemental_files.zip › Supp_Fig_1_HE_alignment_ziag070.pdf]

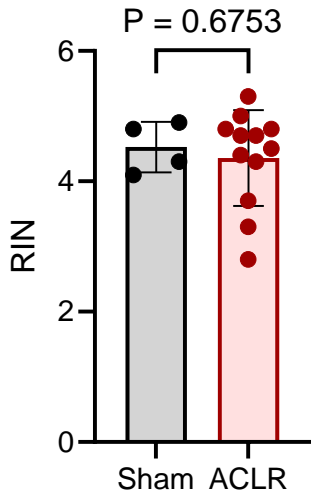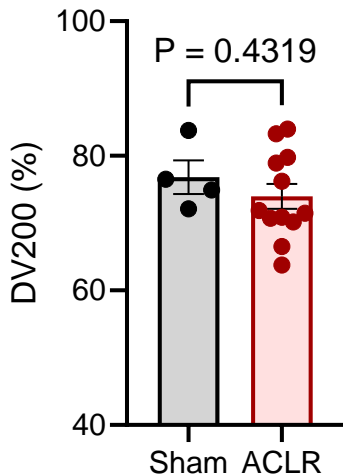

Supplement: ziag070_Supplemental_Files [file ziag070_supplemental_files.zip › Supp_Fig_2_RNA_healthy_injury_ziag070.pdf]
